# Early prediction of left ventricular function improvement in patients with new-onset heart failure and presumed non-ischaemic aetiology

**DOI:** 10.1136/openhrt-2023-002429

**Published:** 2023-08-17

**Authors:** Ze Ming Goh, Wasim Javed, Mubien Shabi, Joel R L Klassen, Christopher E D Saunderson, Jonathan Farley, Melanie Spurr, Erica Dall’Armellina, Eylem Levelt, John Greenwood, Brian Halliday, Sven Plein, Peter Swoboda

**Affiliations:** 1 Leeds Institute of Cardiovascular and Metabolic Medicine, University of Leeds, Leeds, UK; 2 National Heart and Lung Institute, Imperial College London, London, UK; 3 CMR Unit and Inherited Cardiac Conditions Care Group, Royal Brompton and Harefield Hospitals, London, UK

**Keywords:** HEART FAILURE, Magnetic Resonance Imaging, RISK FACTORS

## Abstract

**Objectives:**

To determine baseline characteristics predictive of left ventricular ejection fraction (LVEF) recovery in patients diagnosed with heart failure with reduced ejection fraction (HFrEF) and presumed non-ischaemic aetiology.

**Methods:**

We prospectively recruited patients who were diagnosed with HFrEF (LVEF ≤40%) on echocardiography and subsequently underwent cardiac MRI. Patients were excluded if they had a known history of coronary artery disease (>70% on invasive coronary angiography), myocardial infarction, coronary revascularisation or anginal symptoms. At cardiac MRI assessment, patients were categorised as either ongoing HFrEF or heart failure with improved ejection fraction (HFimpEF, LVEF >40% with ≥10% of absolute improvement). Clinical characteristics were compared between the groups. Logistic regression was performed to identify variables that were associated with LVEF recovery. Optimal cut-offs in QRISK3 score and baseline LVEF for prediction of LVEF recovery were identified through receiver operating characteristic curve analysis.

**Results:**

A total of 407 patients were diagnosed with HFrEF, and 139 (34%) attained HFimpEF at cardiac MRI assessment (median 63 days, IQR 41–119 days). Mean age of the patients was 63±12 years, and 260 (63.9%) were male. At multivariate logistic regression, both QRISK3 score (HR 0.978; 95% CI 0.963 to 0.993, p=0.004) and baseline LVEF (HR 1.044; 95% CI 1.015 to 1.073, p=0.002) were independent predictors of HFimpEF. Among patients with baseline LVEF ≤25%, only 22 (21.8%) recovered. In patients with baseline LVEF 25–40%, QRISK3 score >18% was associated with lack of recovery (HR 2.75; 95% CI 1.70 to 4.48, p<0.001). Additionally, QRISK3 score was associated with the presence of ischaemic late gadolinium enhancement (HR 1.035; 95% CI 1.018 to 1.053, p<0.001).

**Conclusions:**

The QRISK3 score helps identify patients with HFrEF with undiagnosed vascular disease. Patients with either a very low baseline LVEF or a high QRISK3 score have less chance of left ventricular recovery and should be prioritised for early cardiac MRI and close monitoring.

**WHAT IS ALREADY KNOWN ON THIS TOPIC**

- In patients presenting with heart failure and reduced ejection fraction, a total of 21–40% have recovery of left ventricular ejection fraction (LVEF). This can be termed heart failure with improved ejection fraction and is typically defined as patients presenting with LVEF ≤40%, improving to LVEF >40% with ≥10% of absolute improvement.

**WHAT THIS STUDY ADDS**

- In our cohort of patients presenting with heart failure and reduced ejection fraction, a total of 34% had recovery of left ventricular function. Very low LVEF at presentation and high QRISK3 score were associated with reduced left ventricular recovery. It may be possible to use these factors to identify patients unlikely to have recovery of left ventricular function who are at higher risk and might benefit from early cardiac magnetic resonance (CMR).

**HOW THIS STUDY MIGHT AFFECT RESEARCH, PRACTICE OR POLICY**

- There is widespread variability in expertise and availability of CMR. We therefore propose that early CMR should be prioritised to those with either very low LVEF at presentation (LVEF ≤25%) or LVEF 25–40% at baseline with QRISK3 >18%.

## Introduction

Up to 40% of patients with heart failure, particularly those with a non-ischaemic aetiology, undergo reverse remodelling with subsequent improvement in left ventricular ejection fraction (LVEF) after introduction of guideline-directed therapy. This has been termed heart failure with improved ejection fraction (HFimpEF) or heart failure with recovered ejection fraction.^1^ Both of these definitions are typically defined as: (1) a decreased LVEF <40% at baseline; (2) ≥10% absolute improvement in LVEF and (3) a second measurement of LVEF >40%. Factors previously associated with left ventricular (LV) recovery include non-ischaemic aetiology, shorter duration, female sex and absence of myocardial scar on cardiovascular magnetic resonance (CMR).^2^

CMR is increasingly used in the investigation of patients with heart failure. It can provide accurate assessment of cardiac function and characterisation of myocardial tissue with late gadolinium enhancement (LGE) which can provide insight into both the aetiology and prognosis of heart failure. European and US practice guidelines give CMR a class IIa recommendation in the assessment of patients with heart failure, in particular for differential diagnosis of the aetiology and to distinguish between ischaemic and non-ischaemic scar.^3 4^ Even in patients with presumed non-ischaemic cardiomyopathy, ischaemic scar can be found by LGE in a significant proportion.^5 6^ Despite these recommendations, there is widespread variation in practice and many patients with heart failure do not get investigated by CMR largely due to limited availability and expertise.^7^

Patients with HFimpEF have a favourable prognosis compared with patients who do not experience reverse remodelling, although there can be a subsequent decline in LV function particularly in those whose heart failure medications are discontinued.^8 9^

We therefore hypothesised that baseline risk factors could be used to predict which patients with new-onset presumed non-ischaemic cardiomyopathy would have recovery of LV function.

We additionally aimed to identify the proportion of patients with HFimpEF by the time they have CMR and if they could be identified by any baseline clinical or epidemiological factors. We specifically aimed to establish if QRISK3,^10^ a UK prediction algorithm of risk of heart disease and stroke, could identify patients likely to have recovery of LV function.

## Methods

### Study population

**Figure 1:**
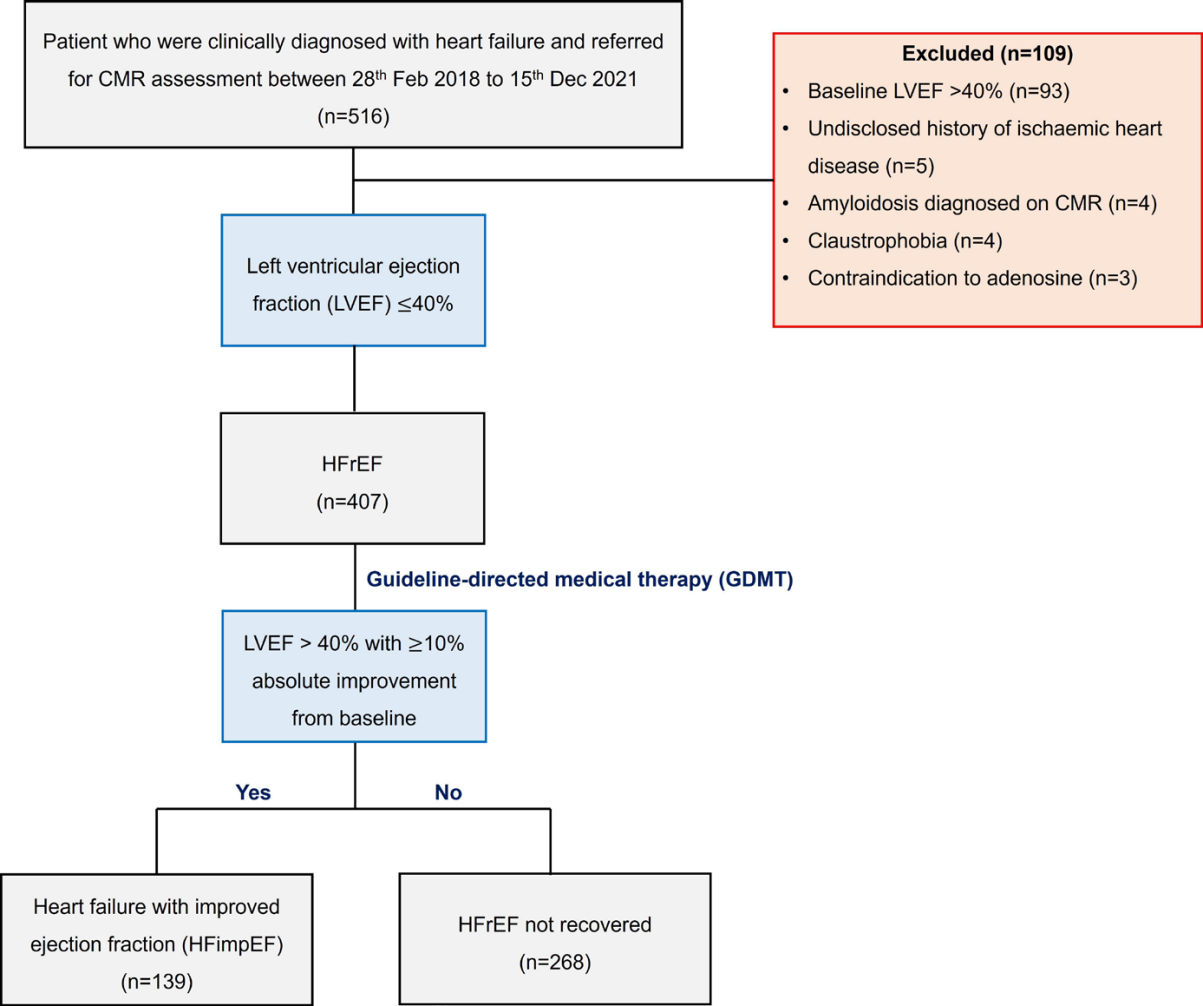
CONSORT diagram. CONSORT, Consolidated Standards of Reporting Trials; HFrEF, heart failure with reduced ejection fraction.

We prospectively recruited patients from the MATCH (MyocArdial Tissue CHaracteristics in patients with heart failure according to glycaemic status) registry who were clinically diagnosed with heart failure at cardiology clinics and referred for CMR assessment between 28 February 2018 and 15 December 2021. Patients were classified to have heart failure with reduced ejection fraction (HFrEF) if found to have a baseline LVEF ≤40% on echocardiography at the time of diagnosis. Patients were excluded if they had a baseline LVEF >40%, known history of coronary artery disease (>70% on invasive coronary angiography), myocardial infarction, coronary revascularisation or anginal symptoms. Other exclusion criteria included hypertrophic cardiomyopathy, amyloidosis, congenital heart disease and advanced renal failure (figure 1). Patients were started on guideline-directed medical therapy (GDMT) after being diagnosed with HFrEF at the discretion of the treating clinician.^3^ As the timing of CMR referral was decided by the clinician, many patients had not completed GDMT uptitration by the time of CMR. HFimpEF was defined as having LVEF of >40% with ≥10% absolute improvement from baseline on the day of CMR assessment.

### Patient characteristics

**Figure 2:**
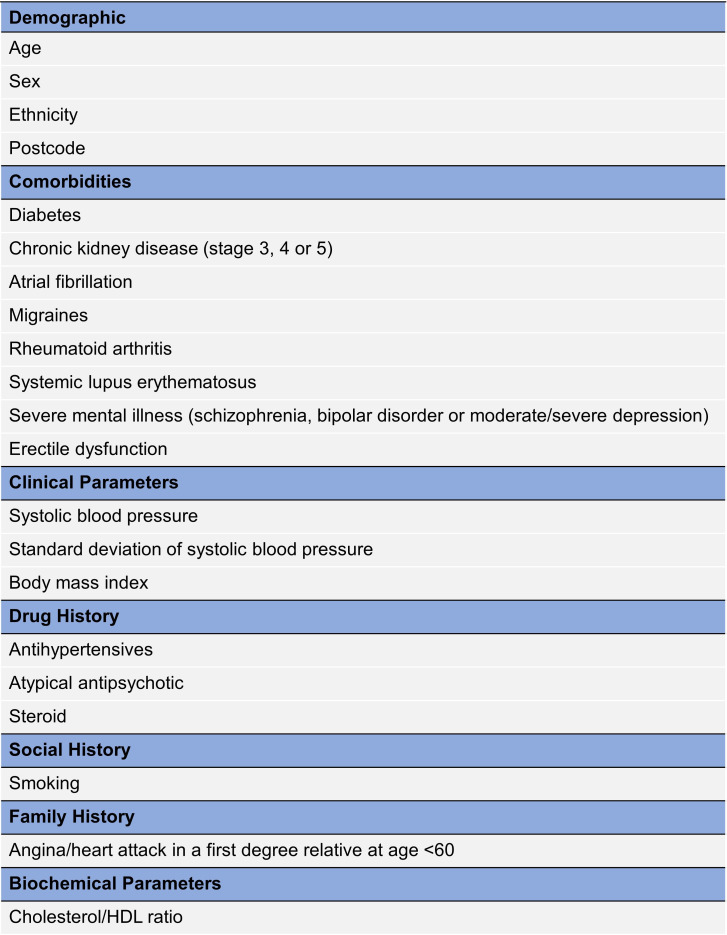
Variables collected in the QRISK3 calculation. HDL, high-density lipoprotein.

Clinical assessment, blood tests and CMR were carried out during one visit. Clinical information including patient demographics, comorbidities, smoking history and medication history was obtained through direct interview and electronic health record. Townsend Deprivation Index was calculated from the patient’s postcode and QRISK3 calculated from this and available clinical data (figure 2). Glycated haemoglobin (HbA1c) was measured on the day of the CMR scan. Echocardiography was reported at site with measurement of LVEF by biplane method whenever possible.

### CMR acquisition

Patients were advised to avoid caffeine intake for 24 hours before CMR assessment. CMR images were acquired in a supine position using a 3T system (Siemens Magnetom Prisma, Erlangen, Germany). The study protocol included cine imaging, stress-perfusion imaging and motion-corrected bright-blood LGE. For stress imaging, adenosine was infused for at least 3 min, at a rate of 140 g/kg/min and if there was insufficient haemodynamic response (increase in heart rate <10 beats/min) or lack of symptomatic response, infused at an increased rate of up to 210 g/kg/min. LGE images were acquired as a short-axis stack, and in four-chamber, three-chamber and two-chamber views. When it was unclear if the enhancement on bright-blood LGE represented ischaemic changes, a dark-blood LGE stack was acquired.

### Image analysis

Image analysis was carried out on the cvi42 software (Circle Cardiovascular Imaging, Calgary Canada). LV volumes were measured by manual segmentation of endocardial and epicardial contours at end-systole and end-diastole.

The presence of LGE was confirmed if enhancement was identified on two orthogonal planes or, where available, on both bright and dark-blood LGE images. Ischaemic LGE was defined as enhancement that involved the subendocardium in a typical coronary distribution. Other patterns of fibrosis were categorised as non-ischaemic with the exception of right ventricular insertion point fibrosis which was deemed not to have fibrosis. Inducible ischaemia was defined as presence of visual perfusion defect affecting >1 myocardial segment on stress perfusion images not matched on rest perfusion or LGE images.

### Statistical analysis

IBM SPSS Statistic V.28 was used for statistical analysis. The patients with HFrEF were divided into the group who achieved HFimpEF and the group who failed to. T-test and Χ^2^ test were used to compare demographic, clinical, echocardiographic and CMR variables between the two groups. Univariate logistic regression was used to evaluate the association between the recovery of LVEF and demographic variables, baseline LVEF and CMR-assessed tissue characteristics. Multivariate logistic regression was performed to assess whether QRISK3 score could predict LV recovery even after correcting for baseline LVEF.

Receiver operating characteristic (ROC) curve analysis and calculation of Youden index were used to identify the optimal cut-off in QRISK3 score and LVEF for prediction of HFimpEF. The prevalence of ischaemic LGE in patients with low and high QRISK3 scores was compared using a t-test. Additionally, the association between the QRISK3 score and ischaemic LGE was assessed through univariate logistic regression.

## Results

### Patients

We included 407 out of 516 patients from the registry. A total of 109 patients were excluded from the study for having baseline LVEF >40% (n=93), undisclosed history of acute coronary syndrome (n=5), amyloidosis (n=4), claustrophobia at CMR assessment (n=4) and contraindication to adenosine (n=3). Out of the 407 patients, 139 patients (34%) attained HFimpEF with GDMT at time of CMR assessment (median 63 days, IQR 41–119 days) (figure 1). Table 1 demonstrates the differences in variables between the patients with HFimpEF and patients who failed to attain recovery of LVEF, including demographics, clinical history, echocardiographic and CMR parameters.

**Table 1:** Results of independent t-test and Χ^2^ test

Clinical parameters	HFimpEF (n=139)	HFrEF not recovered (n=268)	P value
Age (years)	60.9±12.5	63.8±11.6	**0.022**
Sex (male)	67 (51.8)	193 (72.0)	**<0.001**
Body mass index (kg/m^2^)	28.4±5.6	29.8±17.2	0.357
Comorbidities			
Diabetes mellitus, n (%)	19 (13.7)	55 (20.5)	0.089
HbA1c (mmol/mol)	41.2 (10.2)	44.2 (12.3)	**0.009**
Hypertension, n (%)	60 (43.2)	123 (45.9)	0.600
Hypercholesterolaemia, n (%)	39 (28.1)	74 (27.6)	0.924
Cerebrovascular event, n (%)	15 (10.8)	37 (13.8)	0.388
Atrial fibrillation, n (%)	42 (30.2)	105 (39.2)	0.074
Current smoker, n (%)	19 (13.7)	53 (19.9)	0.122
Townsend Deprivation Index	0.4 (3.8)	−0.3 (3.5)	0.060
QRISK3	17.6 (13.9)	22.2 (14.3)	**0.002**
Medications			
Antiplatelets, n (%)	24 (17.3)	50 (18.8)	0.705
Beta-blocker, n (%)	118 (84.9)	229 (86.1)	0.744
Statin, n (%)	54 (38.8)	125 (47.0)	0.117
ACE-I/ARB, n (%)	114 (82.0)	229 (86.1)	0.279
Sacubitril/valsartan, n (%)	8 (5.8)	16 (6.0)	0.916
Aldosterone receptor antagonist, n (%)	42 (30.2)	98 (36.8)	0.183
Diuretic, n (%)	47 (33.8)	145 (54.5)	**<0.001**
Oral anticoagulant, n (%)	40 (28.8)	98 (36.8)	0.329
Echocardiography			
Baseline LVEF (%)	31.4±6.7	28.7±8.7	**<0.001**
Clinical CMR			
LVEDVi (mL/m^2^)	92.9±25.1	122.3±38.1	**<0.001**
LV stroke volume (mL)	90.2±28.7	72.0±23.2	**<0.001**
LVEF (%)	51.8±8.5	31.5±9.5	**<0.001**
LVMi (g/m^2^)	59.5±16.0	73.2±20.3	**<0.001**
RVEDVi (mL/m^2^)	71.5±19.2	79.6±26.3	**<0.001**
RV stroke volume (mL)	74.4±25.0	68.3±23.9	**0.017**
RVEF (%)	54.3±11.2	45.6±13.1	**<0.001**
LAVi (mL/m^2^)	38.5±17.7	46.3±19.6	**<0.001**
Tissue characteristics			
Ischaemic fibrosis, n (%)	10 (7.2)	67 (25.0)	**<0.001**
Number of segments with ischaemic fibrosis (if present), n	3.4±2.5	3.8±2.9	0.614
Inducible ischaemia, n (%)	4 (2.9)	19 (7.1)	0.081
Number of segments with inducible ischaemia (if present), n	3.25±1.7	4.30±3.2	0.534
Non-ischaemic fibrosis, n (%)	33 (23.7)	93 (34.7)	**0.023**
Number of segments with non-ischaemic fibrosis (if present), n	2.03±1.7	2.73±2.3	0.117

P-values set in bold indicate statistical significance.

ACE-I, ACE inhibitor; ARB, angiotensin receptor blocker; CMR, cardiovascular magnetic resonance; HbA1c, glycated haemoglobin; HFimpEF, heart failure with improved ejection fraction; HFrEF, heart failure with reduced ejection fraction; LAVi, left atrium volume index; LVEDVi, left ventricular end-diastolic volume index; LVEF, left ventricular ejection fraction; RVEDVi, right ventricular end-diastolic volume index; RVEF, right ventricular ejection fraction.

### Clinical characteristics, biochemistry and echocardiography

Mean age of the patients was 63±12 years, and 260 (63.9%) were male. The patients with HFimpEF were found to be younger (60.9±12.5 vs 63.8±11.6, p=0.022) and consisted of fewer men (51.8% vs 72.0%, p<0.001) than the patients who failed to attain recovered LVEF. Risk factors or comorbidities, including diabetes mellitus, hypertension, hypercholesterolaemia, previous cerebrovascular events, atrial fibrillation, smoking history and the Townsend Deprivation Index, did not show statistically significant association with HFimpEF. However, when combined in the QRISK3 score, patients with HFimpEF had lower QRISK3 (17.6±13.9% vs 22.2±14.3%, p=0.002) than those who did not recover. There was no difference in GDMT received between the two groups of patients, except patients with HFimpEF were less likely to receive diuretics (33.8% vs 54.5%, p<0.001).

### CMR assessment

There was a lower prevalence of ischaemic (7.2% vs 25.0%, p<0.001) and non-ischaemic fibrosis (23.7% vs 34.7%, p=0.023) in patients with HFimpEF than patients with HFrEF who did not recover. The prevalence of inducible ischaemia was 2.9% in the patients with HFimpEF and 7.1% in the patients with HFrEF who did not recover, respectively (p=0.081). Furthermore, patients with ischaemic LGE had higher QRISK3 scores compared with those without it (26.7 vs 19.2, p<0.001).

### Logistic regression

Table 2 demonstrates the results of the univariate logistic regression analyses. Clinical factors associated with HFimpEF were younger age (HR 0.980; 95% CI 0.964 to 0.997, p=0.023), female sex (HR 2.765; 95% CI 1.805 to 4.236, p<0.001), lower QRISK3 score (HR 0.977; 95% CI 0.962 to 0.992, p=0.003) and higher baseline LVEF (HR 1.045; 95% CI 1.017 to 1.074, p=0.002). Patients with lower HbA1c (HR 0.975; 95% CI 0.955 to 0.995, p=0.017) on the day of CMR were more likely to have had recovery of LV function. Patients with both ischaemic (HR 0.233; 95% CI 0.115 to 0.468, p<0.001) and non-ischaemic fibrosis (HR 0.586; 95% CI 0.368 to 0.932, p=0.024) on CMR were less likely to have recovery of LV function.

**Table 2:** Association between baseline clinical characteristics, CMR findings and incidence of HFimpEF by logistic regression

Variables	Beta	SE	HR	95% CI	P value
Clinical					
Age	−0.020	0.009	0.980	0.964 to 0.997	**0.023**
Female	1.017	0.218	2.765	1.805 to 4.236	**<0.001**
Baseline LVEF	0.044	0.014	1.045	1.017 to 1.074	**0.002**
Diabetes mellitus	−0.489	0.290	0.613	0.348 to 1.082	0.091
HbA1c	−0.025	0.010	0.975	0.955 to 0.995	**0.017**
Hypertension	−0.111	0.211	0.895	0.593 to 1.353	0.600
Hypercholesterolaemia	0.022	0.233	1.022	0.648 to 1.614	0.924
Cerebrovascular event	−0.281	0.326	0.755	0.399 to 1.430	0.389
Atrial fibrillation	−0.397	0.223	0.672	0.434 to 1.041	0.075
Current smoker	−0.447	0.291	0.639	0.362 to 1.130	0.124
Townsend Deprivation Index	0.055	0.029	1.057	0.998 to 1.120	0.061
QRISK3	−0.024	0.008	0.977	0.962 to 0.992	**0.003**
Time between echocardiography and CMR	0.002	0.001	1.002	1.000 to 1.004	**0.029**
Tissue characteristic					
Ischaemic LGE	−1.459	0.357	0.233	0.115 to 0.468	**<0.001**
Number of segments of ischaemic LGE	−0.064	0.126	0.938	0.732 to 1.200	0.610
Inducible ischaemia	−0.946	0.560	0.388	0.129 to 1.165	0.091
Number of segments of inducible ischaemia	−0.142	0.222	0.867	0.562 to 1.339	0.520
Non-ischaemic LGE	−0.535	0.237	0.586	0.368 to 0.932	**0.024**
Number of segments of non-ischaemic LGE	−0.205	0.134	0.815	0.626 to 1.060	0.127

P-values set in bold indicate statistical significance.

CMR, cardiac magnetic resonance; HbA1c, glycated haemoglobin; HFimpEF, heart failure with improved ejection fraction; LGE, late gadolinium enhancement; LVEF, left ventricular ejection fraction.

At multivariate logistic regression, both QRISK3 score (HR 0.978; 95% CI 0.963 to 0.993, p=0.004) and baseline LVEF (HR 1.044; 95% CI 1.015 to 1.073, p=0.002) were identified to be predictors of HFimpEF independent of each other. Both QRISK3 (HR 0.980; 95% CI 0.965 to 0.995, p=0.011) and baseline LVEF (HR 1.039; 95% CI 1.010 to 1.068, p=0.007) remained independent predictors of HFimpEF after correction for time between the baseline echocardiogram and CMR.

Additionally, QRISK3 score was shown to be associated with the presence of ischaemic LGE (HR 1.035; 95% CI 1.018 to 1.053, p<0.001).

### Receiver operating characteristic curve analysis

ROC curves of the QRISK3 score and baseline LVEF predicting HFimpEF were plotted. When predicting failure to achieve HFimpEF, the QRISK3 score had an area under the curve (AUC) value of 0.600 (95% CI 0.543 to 0.658) with an optimal cut-off of 18.055 (Youden index=0.22). On the other hand, when predicting for HFimpEF, the baseline LVEF had an AUC value of 0.585 (95% CI 0.528 to 0.641) with an optimal cut-off of 26.5 (Youden index=0.15).

### Clinical implementation

In order to implement the findings of the ROC analysis, we determined the proportion of patients who developed HFimpEF based on the thresholds baseline LVEF ≤25% and QRISK3 ≤18% (table 3). In patients with LVEF ≤25% at presentation, only 22 (21.8%) had recovery of LV function. In patients with LVEF 25–40%, a QRISK3 score >18% was associated with lack of LV recovery (HR 2.753; 95% CI 1.693 to 4.476, p<0.001).

**Table 3:** Number and percentage of patients attaining HFimpEF according to baseline LVEF and QRISK3

	Baseline LVEF ≤25%, n (%)	Baseline LVEF >25%, n (%)
QRISK3 ≤18%, n (%)	10/41 (24.4)	81/166 (48.8)
QRISK3 >18%, n (%)	12/60 (20.0)	36/140 (25.7)

HFimpEF, heart failure with improved ejection fraction; LVEF, left ventricular ejection fraction.

## Discussion

This study has shown that baseline clinical characteristics can predict short-term recovery of LV systolic function in patients with a new diagnosis of heart failure. In patients with a recent diagnosis of HFrEF referred for clinical CMR to investigate the aetiology of heart failure, by the time of CMR (median 63 days), 34% had recovery of LV function, fulfilling the criteria for HFimpEF. Several patient factors were significantly associated with recovery of LV function including younger age, female sex, lower HbA1c, lower baseline LVEF and lower QRISK3 score. LVEF >25% on baseline echocardiogram and QRISK3 score ≤18% were independently associated with subsequent recovery of LV function.

### QRISK3 to identify patients less likely to have LV recovery

Younger age, female sex and lower HbA1c were associated with the recovery of LV function in patients with HFrEF. Other clinical parameters that were associated with a non-significant trend to prediction of lack of recovery of LV function included history of diabetes mellitus, history of atrial fibrillation and Townsend Deprivation Index. Many of these factors are included in the calculation of the QRISK3 score which explains the strong association between QRISK3 and recovery of LV function.

QRISK3 score was developed from primary care data from many millions of patients in the UK and is designed to predict 10-year risk of incident cases of cardiovascular disease.^10^ Given that silent ischaemic heart disease is strongly associated with lack of recovery of LV function and QRISK3 is predictive of incident cardiovascular disease, it is intuitive that QRISK3 can be used to identify those least likely to have recovery of LV function. The predictive potential of QRISK3 emphasises the fact that there are shared risk factors and likely disease mechanisms between presumed non-ischaemic HFrEF and vascular disease.

Using QRISK3 to identify those less likely to have recovery of LV function is attractive because it is widely and freely available; both patients and doctors are already familiar with its use (particularly regarding statin prescribing); and it has been derived and validated in the UK population.

### Natural history of HFimpEF

In our study, 34% of patients achieved HFimpEF, which is comparable with other published contemporary studies that report a rate of recovery between 21% and 40%.^11–13^ However, more direct comparisons between studies are difficult due to the differences in baseline characteristics, GDMT usage, interval between scans and even variations in the definition of LV recovery. Patients with HFimpEF still have a degree of morbidity and chance of future decline in LV function. They should continue medical therapy where possible.^8 14^ CMR was performed relatively early in our cohort (median 63 days, IQR 41–119 days) and if the scans were performed later, it is possible that a higher proportion of patients would attain HFimpEF.

The prognosis of HFimpEF compared with HFrEF is significantly improved with a significant reduction in all-cause mortality ranging from 45% to 76%.^11 12 15^ Therefore, we postulate that early CMR may be beneficial for patients who are unlikely to attain recovery of LV function. Early CMR may assist physicians in identifying patients who are less likely to recover and may require more intensive medical therapy and even early implantable cardioverter defibrillator implantation.

### CMR phenotype in patients without recovery of LV function

In addition to clinical parameters, several CMR findings were associated with lack of recovery of LV function. Most prominently, the presence of ischaemic or non-ischaemic scar on LGE CMR was associated with a lack of recovery of LV function with an HR of 4.29. In this cohort, no patients had symptoms of angina, history of recognised myocardial infarction or prior revascularisation, but 19% of patients had ischaemic scar, indicating unrecognised myocardial infarction. The presence of ischaemic scar is well recognised to be a predictor of adverse outcomes both in patients with history and symptoms of ischaemic heart disease,^16 17^ as well as in asymptomatic patients.^18 19^

The presence of non-ischaemic fibrosis was also significantly associated with lack of recovery of LV function although with a lesser HR of 1.71. These results are consistent with previous reports in which the presence and extent of non-ischaemic fibrosis in dilated cardiomyopathy were associated with outcomes including arrhythmia, heart failure and mortality.^20 21^

Although both ischaemic and non-ischaemic fibrosis are associated with lack of LV recovery, it is not clear whether they play a causative role or are a marker of irreversible processes.

The CMR phenotype of patients without recovery of LV function also included increased LV, right ventricular and atrial volumes and poorer right ventricular function. However, as we did not have paired CMR data at baseline, it is not clear if these factors could be predictive or are merely associated factors with lack of recovery.

### Clinical translation

CMR is recommended in National Institute for Health and Care Excellence and European Society of Cardiology (ESC) and American Heart Association/American College of Cardiology Foundation/Heart Failure Society of America guidelines,^3 4 22^ particularly in those with suboptimal echocardiographic imaging or suspected myocardial tissue disease. In patients with dilated cardiomyopathy, the ESC guidelines give a IIa recommendation to identify occult ischaemic myocardial damage using CMR.^3^

There are practical challenges in implementing these recommendations relating to scanner availability and CMR expertise. The 2018 British Society for Cardiovascular Magnetic Resonance Survey revealed wide variance in the clinical use and waiting times in the UK with a >10-fold difference between the regions with the highest and lowest-level CMR activity. Our findings could be used to guide physicians in identifying patients who might benefit from early CMR and those in whom CMR could be delayed allowing recovery of LV function. Where CMR resources are limited, we propose that in patients with suspected non-ischaemic HFrEF, early CMR should be prioritised to:

1. Patients with very low LVEF (≤25%).
2. Patients with LVEF 25–40% but QRISK3 score >18%.

### Limitations

The patients in our study were real-world patients with heart failure and there was variety to the extent in which they were being treated with optimal medical therapy. The majority were being treated with ACE inhibitors and beta-blockers, but use of mineralocorticoid receptor antagonists was modest. It is possible with increased prescribing of heart failure medications there would have been a greater proportion of HFimpEF.

Echocardiographic data were not analysed in a core laboratory, but on-site measurement of LVEF was used. However, it was not the purpose of this study to accurately compare LVEF between the initial echocardiogram and subsequent CMR but rather identify clinical factors which might identify patients unlikely to have recovery of LV function. We have chosen to use a categorical definition of HFimpEF. The association between baseline LVEF and recovery may have been diminished if LV recovery was defined as a continuous variable.^2^ CMR was performed in a single centre but referrals were taken from multiple hospitals in the region allowing for wide variations in treatment and patient factors, including social deprivation.

## Conclusions

In this cohort of patients presented with HFrEF, 34% had recovery of LV function. A very low LVEF at presentation and high QRISK3 score were both predictive of patients with a lack of LV recovery. It may be possible to use these factors to identify patients unlikely to have recovery of LV function who might benefit from early CMR and close monitoring.

## Data Availability

Data are available upon reasonable request.
